# Study on Quality Detection Methods for Table Grapes Based on Spectral and Imaging Information

**DOI:** 10.3390/s26082343

**Published:** 2026-04-10

**Authors:** Licai Chen, Zheng Zou, Shulin Yin, Jiang Luo, Xinhai Wu, Huaichun Xiao, Jing Xu

**Affiliations:** 1 Institute of Agricultural Engineering, Jiangxi Academy of Agricultural Sciences, Nanchang 330200, China; clc-1985@163.com; 2 Jiangxi Key Laboratory for Mass Spectrometry and Instrumentation, East China University of Technology, Nanchang 330013, China; 17872670616@163.com (Z.Z.); 15880019453@163.com (S.Y.); luo19970702@163.com (J.L.); 13197913903@163.com (X.W.); 17726963530@163.com (J.X.); 3 Engineering Research Center for Seismic Disaster Prevention and Engineering Geological Disaster Detection of Jiangxi Province, East China University of Technology, Nanchang 330013, China

**Keywords:** table grape, quality detection, hyperspectral imaging, SSC

## Abstract

The increasing demand for high-quality grapes necessitates rapid and objective quality assessment methods to overcome the limitations of traditional subjective and destructive techniques. This study investigated the feasibility of using hyperspectral imaging combined with machine learning for non-destructive quality evaluation of fresh grapes. Hyperspectral data were acquired from four table grape varieties (“Rose”, “Yongyou”, “Xiahei”, and “Jumbo”), and their Soluble Solids Content (SSC) was measured, which varied significantly among varieties. We extracted texture features using the Gray-Level Co-occurrence Matrix (GLCM) from images at key wavelengths, which were a combination of those selected by the Successive Projections Algorithm (SPA) and sensitive wavelengths. Comparative models for variety classification (qualitative) and SSC prediction (quantitative) were built using Extreme Learning Machine (ELM), Convolutional Neural Network (CNN), and Partial Least Squares (PLS) with full-range spectra and texture features as inputs. The results showed that the ELM model using full-range spectra was superior for both tasks, achieving a classification accuracy of 97.56% and, for SSC prediction, an $R_{p}^{2}$ of 0.75 and an RMSEP of 0.81. Notably, CNN models also showed considerable robustness. Our findings confirm that combining hyperspectral imaging with machine learning is a viable strategy for fresh grape quality assessment.

## 1. Introduction

Table grapes are one of the most popular summer fruits among consumers, valued not only for their sweet and juicy taste but also for being rich in vitamins and bioactive compounds, which contribute to enhancing immunity and delaying senescence [1,2]. With the rapid expansion of grape cultivation areas, consumer demands for fresh fruit quality have progressively increased, making quality assessment particularly crucial for the industry’s development. Grape quality primarily comprises external and internal attributes; the former includes characteristics such as color, size, and hardness, while the latter mainly involves Soluble Solids Content (SSC), the sugar–acid ratio, and phenolics [3]. Currently, the quality assessment of table grapes predominantly relies on conventional methods. Manual inspection, which depends on human experience to differentiate visible features like color, shape, and firmness, suffers from high subjectivity and low accuracy. Meanwhile, the determination of indicators such as SSC and the sugar–acid ratio is mainly conducted through physicochemical analysis, which is characterized by long analytical cycles and high costs [4,5]. Therefore, exploring faster, non-destructive, and reliable detection methods has become imperative to ensure the sustainable development of the industry and to increase the income of growers.

In recent years, the rapid advancement of technology has led to the widespread adoption of non-destructive testing (NDT) techniques, which are favored for being non-polluting, non-damaging, and highly efficient [6,7]. As an effective modern NDT method, hyperspectral imaging (HSI) technology integrates the features of spectroscopy and imaging, enabling the simultaneous acquisition of an object’s chemical attributes and spatial information. The collected data contains multiple information bands corresponding to different characteristic frequencies [8]. HSI has been widely applied in fruit quality detection, including the assessment of hardness, physicochemical indicators, maturity, diseases, and defects [9,10,11,12,13,14]. The identification of table grape varieties is a crucial basis for the research and development of grape germplasm resources, and HSI has achieved notable results in this area. For instance, Nogales-Bueno et al. acquired HSI of four grape varieties in the 900–1700 nm range, collected their anthocyanin profiles and colorimetric images, and developed stepwise linear discriminant analysis models [15]. A comparison revealed that the HSI-based method achieved a higher correct discrimination rate for grape varieties. Diago et al. used visible and short-wave near-infrared HSI to discriminate the pigment fingerprints of eight different grape berry varieties, establishing a qualitative model with multivariate Partial Least Squares that yielded a coefficient of determination ($R_{p}^{2}$) of 0.86 [16].

The content of indicators such as SSC in table grapes directly influences their flavor and is a critical metric for quality. Several scholars have conducted research on the internal quality of table grapes. Baiano et al. studied seven table grape varieties, acquiring their HSI reflectance to build prediction models for pH, total acidity, and SSC content based on Partial Least Squares Regression (PLSR); all three models achieved $R_{p}^{2}$ values above 0.8 [17]. Gomes and Gao et al. both acquired HSI data of table grapes in the 380–1100 nm wavelength range and developed models to estimate SSC using various preprocessing and modeling techniques. A comparison showed that the model built by Gao et al. yielded a higher $R_{p}^{2}$ value, exceeding 0.97 [18,19]. Gao et al. collected HSI of 360 grapes, measured their total acidity and hardness, and after spectral preprocessing and feature variable extraction, established PLSR-based prediction models. The results indicated that the optimal prediction models for both indicators had $R_{p}^{2}$ values above 0.9 [20]. Additionally, spatial features of images—such as texture, color, and morphology—have increasingly demonstrated significant value in fruit quality assessment. In recent years, researchers have specifically extracted visual features from conventional or hyperspectral images and successfully applied them to the prediction of Soluble Solids Content (SSC) and variety classification in agricultural products like grapes. These studies confirm the effectiveness of image data in reflecting the correlation between the external phenotype and internal quality of fruits [21,22]. A comprehensive analysis of the literature reveals a limitation of using single-modality approaches; studies on table grape quality detection using HSI often fail to perform detection from both spectral and imaging perspectives simultaneously, thus not fully leveraging the “Spectral Image Integration” characteristic of HSI. Furthermore, they typically focus on a single aspect, such as variety identification or the prediction of a single physicochemical value.

HSI of table grapes not only reflects information on the overtone and combination vibrations of hydrogen-containing groups within the organic molecules of their physicochemical components but also highlights internal structural features. The differences in spatio-spectral data among different samples lay the foundation for their quality detection [23]. This study selected four table grape varieties—”Rose”, “Yongyou”, “Xiahei”, and “Jumbo”—as research subjects. Data were acquired using an HSI system, and the differences in their SSC content were simultaneously measured and analyzed. From both spectral and imaging perspectives, this work analyzes the relationship between the SSC of table grapes and their spatio-spectral response characteristics to reveal the underlying mechanism of HSI detection for grape quality. The Successive Projections Algorithm (SPA) was used for data dimensionality reduction. By developing qualitative and quantitative models for table grapes using machine learning and deep learning methods, this study explores a detection method for table grape quality based on both spectral and image data. The findings are intended to provide a basis for establishing a non-destructive detection system for fresh grape quality and to support the development of the grape industry.

## 2. Materials and Methods

### 2.1. Sample Harvesting and Processing

**Figure 1:**
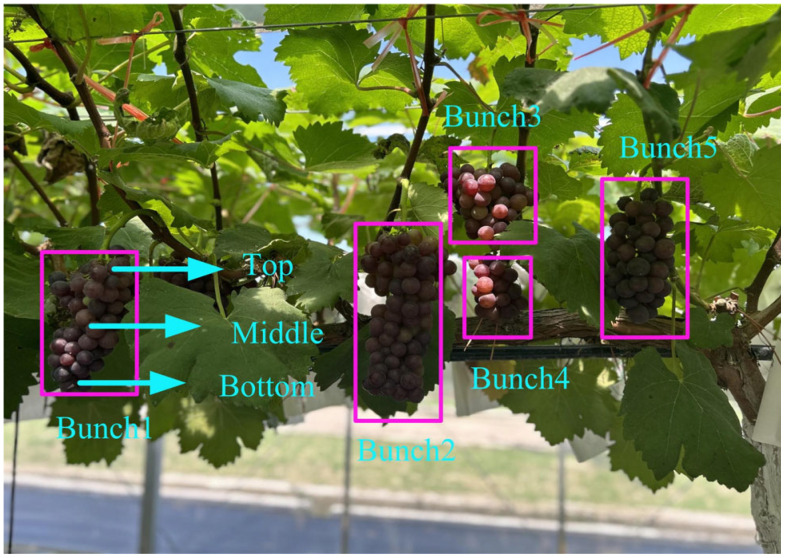
Example of the sampled grape cluster.

The experimental samples were procured from a vineyard situated in Ji’an City, Jiangxi Province (26°57′50″ N, 114°58′47″ E), characterized by a humid, mild, and cool climate [24]. Under the guidance of horticulturists, we harvested four grape varieties—“Jumbo”, “Rose”, “Xiahei”, and “Yongyou”—which appear visually similar and are difficult to distinguish by the naked eye. For each variety, five grapevines were randomly selected, and from each vine, five clusters of grapes were chosen at random. To maintain the physiological integrity of the table grapes, scissors were used to cut at the grape stems at the upper, middle, and lower positions of each cluster, retaining the stems to ensure their integrity, resulting in a total of four samples: one from the upper part, one from the lower part, and two from the middle, as illustrated in Figure 1. Each grapevine yielded a total of 20 table grapes, accumulating to 100 grapes per variety and a combined total of 400 table grapes from the four varieties, meeting the minimum requirement for subsequent SSC measurements. All table grapes were harvested on the same day, and the collected samples were promptly transported to a laboratory maintained at a temperature of 22 ± 2 °C and a humidity of 50% for washing with deionized water and drying. This procedure aimed to enhance the reliability of the data collection. The samples were then appropriately packaged in self-sealing bags and labeled. All samples were stored at 4 °C until the completion of all data collection.

### 2.2. Data Acquisition and Calibration

**Figure 2:**
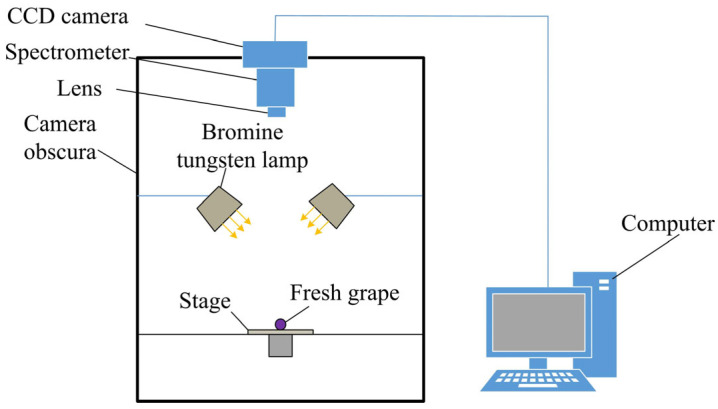
Hyperspectral imaging system structure block diagram.

The HSI system used to scan the four types of table grapes consists of several components, including a light source, a CCD camera, a spectrometer, a sample stage, a dark chamber, and a computer. The structural block diagram of the system is illustrated in Figure 2. The light source comprises two sets of four tungsten halogen lamps (ranging from 350 to 2500 nm, with a power of 20 W) fixed to the inside of the dark chamber to provide illumination for the system. The core components for image acquisition include a CCD camera (C8484-05G, Hamamatsu Photonics, Hamamatsu, Japan) with a resolution of 1344 × 1024 pixels, a spectrometer (ImSpector V10E, Spectral Imaging Ltd., Oulu, Finland) that operates within the wavelength range of 398~1015 nm, and a lens (V23, Spectral Imaging Ltd., Finland). The spectrometer has a spectral channel of 256 and utilizes an InGaAs detector. The dark chamber is used to eliminate the influence of external light, thus enhancing the reliability of the data collected. A movable sample stage is employed to hold the items to be tested. The SpecView 2.5 software, operating on the computer, facilitates system control, enabling the scanning of samples and the storage of data.

**Figure 3:**
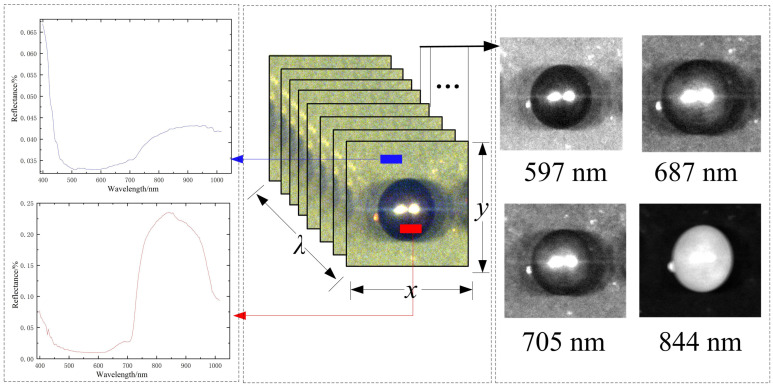
Three-dimensional diagram of table grape hyperspectral image.

Before scanning the samples, the system was turned on and warmed up for 30 min to avoid interference from unrelated factors. The relevant parameters were configured in the SpecView control software, with a spectral resolution of 2.8 nm and an exposure time of 0.08 s. The stage’s forward and backward speeds were set to 1.5 cm/s and 3.0 cm/s, respectively. The samples were laid flat on the surface of the stage, and reflection images in the wavelength range of 398~1015 nm were collected. The images form a three-dimensional data cube, containing spectral information at specific pixels and image information at certain wavelengths, as illustrated in Figure 3.

To eliminate the effects of system dark current, we performed a black-and-white correction on the raw images after collecting all sample images [25,26,27]. First, we covered the camera lens with a lens cap to capture a dark reference image. Next, we removed the lens cap and acquired an image of a polytetrafluoroethylene standard white reference board. Finally, we calculated the corrected HSI of the samples, denoted as R_λ_, using Equation (1).

(1) $$R_{\lambda }=\frac{R_{0(\lambda )}-D_{\lambda }}{W_{\lambda }-D_{\lambda }}$$

In this equation, R_0(λ)_ represents the intensity of reflected light at wavelength λ, while D_λ_ and W_λ_ denote the dark current and white light reflectance at the same wavelength, respectively. The HSI images region of interest (ROI) of four table grapes were artificially selected in ENVI 5.3 software, and the average ROI spectral signatures of each grape were used for subsequent data analysis. When selecting the ROI, care was taken to avoid areas of reflection and the stem area to prevent spectral overfitting and spectral interference from the stem. This approach not only reduces the data scale, thereby enhancing computational efficiency, but also minimizes the interference of non-ROI factors on the results [28].

### 2.3. Table Grape SSC Measurement

The SSC of table grape samples was determined using a digital saccharimeter (PAL-1, ATAGO, Tokyo, Japan). The instrument was initially calibrated with deionized water, and the zero point was adjusted accordingly. Subsequently, 2–3 drops of juice extracted from the sample’s skin were applied to the measurement prism. The “START” button was then engaged to initiate the SSC reading. Each sample underwent triplicate measurements, and the average SSC value was recorded. This procedure was repeated until all samples were analyzed.

### 2.4. Table Grape Variety Labeling and Dataset Segmentation

A small number of samples were damaged to varying degrees during storage. To ensure that subsequent models remain unaffected, we excluded a total of 55 damaged samples from the four varieties of table grapes. This included 13 samples of “Jumbo”, 19 samples of “Rose”, 7 samples of “Xiahei”, and 16 samples of “Yongyou”. The remaining samples totaled 345, specifically comprising 87, 81, 93, and 84 samples from these varieties, respectively.

For qualitative modeling analysis, labels 1, 2, 3, and 4 were randomly assigned to represent the four table grape cultivars “Jumbo”, “Rose”, “Xiahei”, and “Yongyou”, respectively. Division thresholds T were sequentially set at 1.5, 2.5, and 3.5. The remaining 345 samples were randomly partitioned into training and prediction sets following an approximate 3:1 ratio, as detailed in Table 1.

### 2.5. Data-Processing Methodology

#### 2.5.1. Extreme Learning Machine

**Figure 4:**
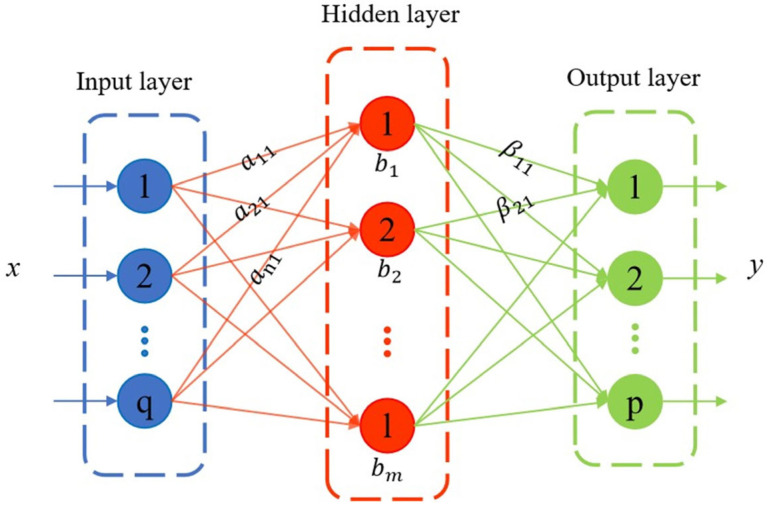
Extreme Learning Machine structure diagram.

Extreme Learning Machine (ELM) is a new single hidden-layer feed-forward neural network model, which consists of three parts: input layer, hidden layer, and output layer, and its structure is shown in Figure 4 [29].

The training stages involve the use of randomly selected weights for the input layer and implicit layer biases, alongside minimizing the loss function to determine the weights of the output layer. This approach allows for rapid and effective generalization. With its fewer tuning parameters, excellent learning capabilities, and high applicability, the mathematical model can be represented by Equation (2) [30].

(2) $$y_{i}=\sum_{j=1}^{m}\beta_{j}g(a_{i}x_{i}+b_{i})$$

In this equation, m denotes the number of hidden layer nodes, g(x) represents the activation function, and αi and bi are the weights and biases of the hidden layer, respectively, which are generated randomly during training. Additionally, βj refers to the output weight, a parameter that requires optimization.

#### 2.5.2. Convolutional Neural Network

Convolutional Neural Networks (CNNs), a principal deep learning architecture for processing and analyzing grid-structured data, feature a core structure comprising input layers, convolutional layers, pooling layers, fully connected layers, and output layers. This architecture offers inherent advantages, including local connectivity and weight sharing, which collectively achieve parameter dimensionality reduction while enabling data-adaptive feature extraction through autonomous learning mechanisms optimized for specific data characteristics [31].

#### 2.5.3. Partial Least Squares

Partial Least Squares (PLS), a multivariate statistical analysis method, transforms high-dimensional variables into a reduced set of latent variables (LVs) to establish linear relationships between multivariate signals and target values. Unlike conventional dimensionality reduction techniques, PLS optimizes LVs extraction by simultaneously maximizing explained variance in predictors and covariance with response variables. This dual optimization enables robust analysis of spectral data exhibiting multicollinearity, high noise levels, and high dimensionality [32].

### 2.6. Model Evaluation Indicators

The most important evaluation index of the model in the qualitative analysis is the discriminant correct rate; the larger its value, the better the model prediction effect, calculated as Equation (3).

(3) $$\mathrm{Accuracy}\;\mathrm{Rate}=\frac{\mathrm{Number}\;\mathrm{of}\;\mathrm{Correctly}\;\mathrm{Classified}\;\mathrm{Samples}\;}{\mathrm{Total}\;\mathrm{Number}\;\mathrm{of}\;\mathrm{Predicted}\;\mathrm{Samples}}\times 100\%$$

The model merit assessment is also carried out by combining the $R^{2}$ and the root mean square error (RMSE). The larger the former is, the closer the relationship between the training set and the prediction set is, and the latter reflects the degree of dispersion between the two, the smaller the value is, the better the model performance, whose formulas are shown in Equations (4) and (5), which are also used as the most commonly used evaluation indexes in quantitative analysis.

(4) $$R^{2}=1-\frac{\sum_{i=1}^{n}{\left(y_{i}-{\hat{y}}_{i}\right)}^{2}}{\sum_{i=1}^{n}{\left(y_{i}-{\bar{y}}_{n}\right)}^{2}}$$

(5) $$RMSE=\sqrt{\frac{1}{n-1}{\sum_{i=1}^{n}\left(y_{i}-{\hat{y}}_{i}\right)}^{2}}$$

where $y_{i}$ is the actual value of the ith sample, ${\;\hat{y}\;}_{i}$ is the predicted value of the ith sample, ${\bar{y}}_{n}$ is the average value of the samples, and n is the number of samples.

## 3. Results

### 3.1. HSI Spectral Characterization of Table Grapes

#### 3.1.1. Spectral Extraction and Characterization of ROI in Table Grapes

**Figure 5:**
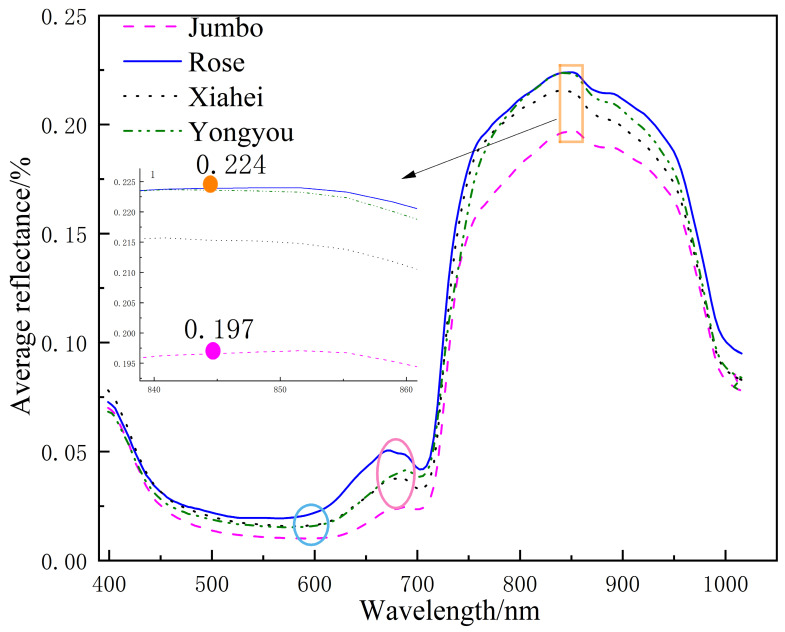
Average ROI spectrum of 4 kinds of table grapes.

The average reflectance spectra of four table grape varieties—”Jumbo”, “Rose”, “Xiahei”, and “Yongyou”—were calculated from their ROI, as illustrated in Figure 5. The results indicate that the average spectral curves of the four grape varieties exhibit similar shapes, characterized by two peaks and two troughs. However, there are noticeable differences in reflectance among the different grape varieties. The “Rose” variety shows the highest reflectance, while the “Jumbo” variety demonstrates the lowest. The reflectance of “Xiahei” and “Yongyou” grapes is relatively close to each other, with slight variations except around 844 nm. The wavelength range of 400–680 nm corresponds to the pigment absorption bands of table grapes, which may vary due to differences in maturity levels among the varieties, ultimately affecting the skin color and, consequently, the spectral absorption properties [33]. The reflectance in the 710–980 nm range may vary due to differences in the chemical compounds present in the grapes, resulting in inconsistent reflectance values [34,35]. Notably, a significant reflectance peak is observed near 844 nm, primarily attributed to the second overtone stretching vibrations of C-H groups. In this region, the reflectance for “Rose” grapes reaches a maximum of 0.224%, while “Jumbo” grapes show a minimum reflectance of 0.197%. Additionally, a less pronounced reflectance peak is observed around 680 nm, which is related to the vibrations of C-H groups and the fourth overtone absorption [36].

**Figure 6:**
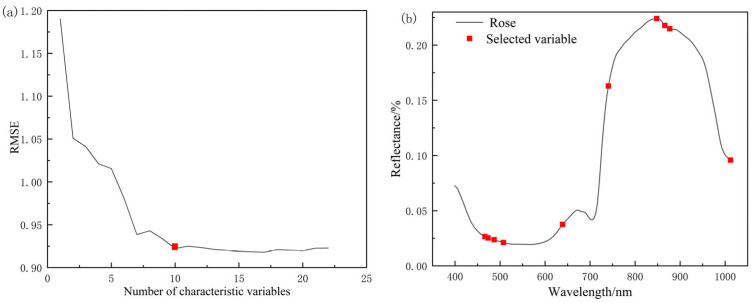
ROI spectral wavelength of table grapes extracted by SPA. (**a**) Variation of RMSE with the number of characteristic variables; (**b**) Distribution of the 14 selected characteristic wavelengths on the average spectral curve.

Hyperspectral data of table grapes typically exhibit strong inter-band correlation and high dimensionality. To eliminate multicollinearity, reduce information redundancy, and improve modeling efficiency [37], the Successive Projections Algorithm (SPA) was employed in this study for characteristic wavelength selection. The number of wavelengths to be extracted by SPA was configured within a range of 5 to 30. As illustrated in Figure 6a, when the number of extracted wavelengths reached 10, the root mean square error (RMSE) decreased to 0.92, and the downward trend subsequently leveled off. This indicates that these 10 wavelengths encompass the majority of the effective information of the table grapes. Figure 6b presents the distribution of the 10 wavelengths selected by SPA, specifically 466.58, 473.38, 487, 507.5, 638.85, 740.88, 848.05, 866.10, 876.92, and 1011.95 nm. Subsequently, these results were merged with the sensitive wavelengths previously extracted in Section 2.2. Given that an interval of at least 10 nm between adjacent wavelengths is required to yield distinct visual differences in grayscale images, two overly proximate wavelengths (473.38 and 848.05 nm) were excluded. Ultimately, 12 characteristic wavelengths were retained for the subsequent modeling process.

#### 3.1.2. Table Grape Image Characterization and Texture Feature Extraction

**Figure 7:**
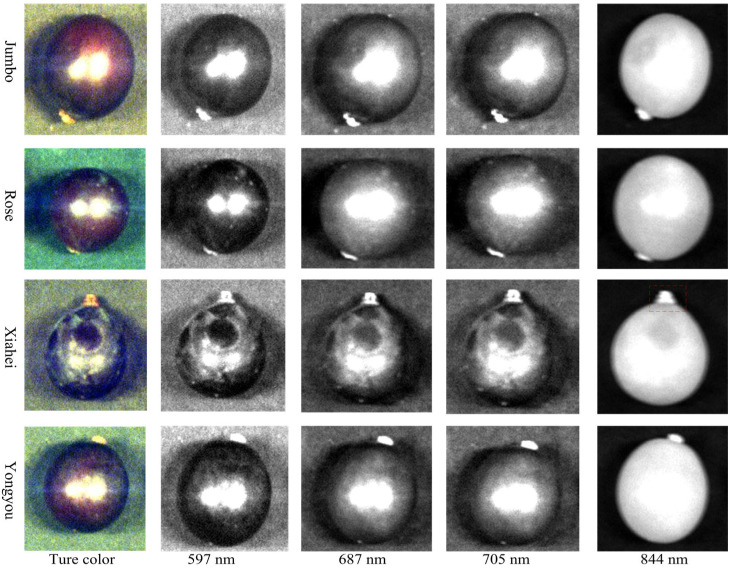
Gray level of different sensitive wavelength points of 4 kinds of table grapes.

Based on the spectral characteristic analysis of the ROI of the samples, the grayscale images that best reflect the characteristic information of table grapes were extracted from their HSI, as shown in Figure 7. From top to bottom, the grape varieties are “Jumbo”, “Rose”, “Xiahei”, and “Yongyou” respectively. From left to right, the corresponding wavelength points are 597, 687, 705, and 844 nm. It was found that the four types of table grapes are generally similar in appearance. In the grayscale images at 597 and 844 nm wavelengths, the outlines of the samples are clear, and in the latter, the boundary between the samples and the background is obvious. The “Xiahei” grapes have a pointed and extended shape at the stem—connecting part, as shown by the red dotted frame in the figure, which is particularly evident in the grayscale image at 844 nm wavelength.

**Figure 8:**
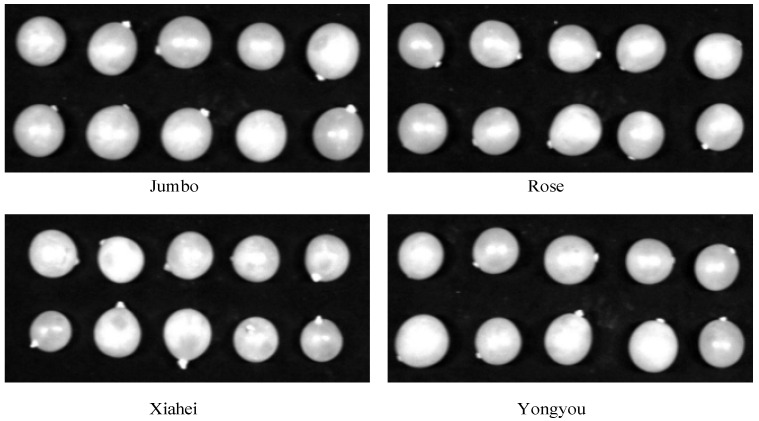
Gray-scale image of 844 nm characteristic wavelength of 4 fresh grapes.

A texture feature analysis was carried out on the grayscale image of a randomly selected wavelength point (844 nm) from the 12 wavelength points, while actively avoiding the stem area, as shown in Figure 8. For each variety, ten samples were randomly selected as a group. It was found that the outlines of table grapes are distinct. The berries of “Jumbo” are relatively large, while the “Xiahei” grapes have a more pointed shape. It is difficult to distinguish them simply by appearance. Therefore, texture features were extracted to establish a model for variety classification and SSC prediction.

Texture features can describe in detail the surface structure and patterns of regions in an image. Their complexity and regularity provide key clues for the classification of different substances and the quality detection. The GLCM is an effective texture analysis technique based on the second-order statistics of the co-occurrence matrix. It mainly measures the joint distribution probability of two pixels at a specific gray level in a specified direction and at a certain distance. Since the reflectance of the samples within the effective wavelength range typically falls within a reasonable interval, whereas the reflectance in glare regions approaches saturation and is significantly higher than normal levels, an adaptive reflectance threshold was established. Pixels with reflectance values exceeding the normal range in each band image were subsequently identified and eliminated. Building upon this, the inter-pixel distance parameter d was set to 1, and Gray-Level Co-occurrence Matrices (GLCM) were calculated across four directions: 0°, 45°, 90°, and 135°. Four commonly used texture descriptors—energy, contrast, correlation, and homogeneity—were selected to represent the texture information [38]. The values of these descriptors were averaged across the four directions, ultimately yielding four feature variables extracted from each grayscale image to serve as inputs for the models.

### 3.2. Differential Analysis of SSC Content in Table Grapes

An ANOVA analysis of SSC was conducted using SPSS 19 software to evaluate the significance of differences among the four grape varieties, with the results summarized in Table 2. The maximum SSC value observed was 21.30° Brix for the “Yongyou” variety, while the minimum was 14.25° Brix for the “Jumbo” variety. The highest average SSC value was recorded for “Yongyou” at 19.65° Brix, whereas the lowest average was for “Xiahei” at 16.23° Brix. These variations may be attributed to factors such as maturity, color, and varietal characteristics.

**Figure 9:**
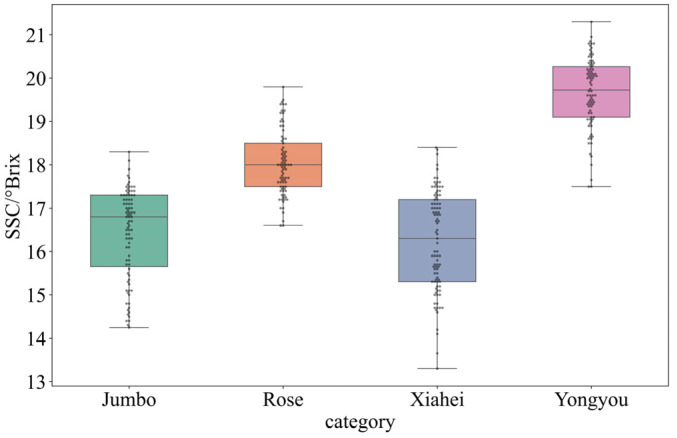
Box line–bee colony diagram of SSC content distribution in table grape.

Among them, F = 230.48 and *p* < 0.05, indicating that there are significant differences in the SSC among these four types of table grapes. A box-swarm plot of the SSC content distribution of the four types of table grapes was drawn, as shown in Figure 9. It was found that the SSC content of “Xiahei” table grapes is more dispersed compared with the other three types, and the SSC content of “Yongyou” table grapes is higher than that of the other three types. There are overlapping parts in the SSC content of the four types of table grapes. It is impossible to distinguish the varieties of table grapes based on their SSC content, and it cannot accurately reflect their biochemical characteristics. However, it provides a theoretical basis for the quality detection of table grapes. Therefore, in the subsequent work, a quality detection model for table grapes was established by combining their spectral and image texture features.

### 3.3. Modeling and Analysis of Table Grape Variety Discrimination

Based on the analysis of the average spectra extracted from the ROI of the table grapes, no obvious random noise signals were observed at the extremities (head and tail ends) of the wavelength range (398–1015 nm). Concurrently, models were established using both the “full-band” data and the data with the “head and tail edge bands removed” to compare their predictive performances. The results revealed that the difference in efficacy between the two models was negligible, indicating that the edge bands did not negatively interfere with the full-band model. Consequently, to retain as much sample information as possible, the full-band data were utilized as the basis for subsequent data processing.

**Figure 10:**
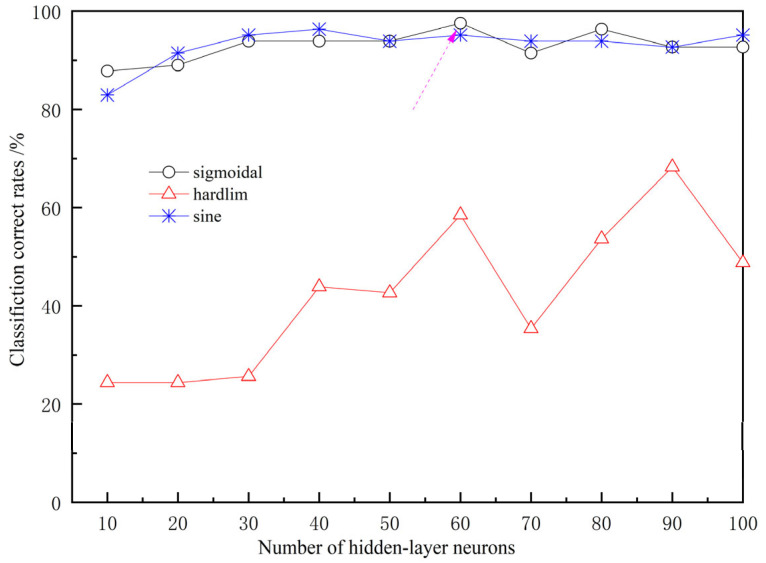
The relationship between the accuracy of three functions and the number of nodes in the hidden layer.

In this paper, an ELM model was established with the ROI spectra of table grapes as the input and combined with their variety label numbers. The initial number of hidden layer nodes was set to 10 and increased to 100 in steps of 10. Three functions, namely sigmoidal, hardlim, and sine, were used as activation functions for learning, respectively. The connection weights between the input layer and the hidden layer and the biases of the hidden layer nodes were randomly generated. By adjusting the activation functions and the number of hidden layer nodes, the discrimination accuracy rate of samples in the prediction set was used as the evaluation criterion to measure the selection quality of activation functions and the number of hidden layer nodes. The discrimination accuracy rates of the ELM model for samples in the prediction set under different activation functions and numbers of hidden layer nodes are shown in Figure 10.

It can be seen that the discrimination accuracy rate of the ELM model with the hardlim activation function is significantly lower than that of the other two functions. Moreover, the discrimination accuracy rates show an upward trend as the number of hidden layer nodes increases. Among them, when the sigmoidal function is used as the activation function, and the number of hidden layer nodes is 60, the discrimination accuracy rate of the ELM model reaches the highest value of 97.56% and then shows fluctuations and a slight decline. Although the peak discrimination accuracy rate of the ELM model with the sine function as the activation function is 96.34%, it is lower than that of the former. Therefore, in the subsequent qualitative ELM model, the sigmoidal function was used as the activation function, and the number of hidden layer nodes was set to 60.

In the experiment, the 1D-CNN qualitative model included two convolutional layers, two maximum pooling layers, a fully connected layer, and an activation function. The number of convolutional kernels in the convolutional layers was 16 and 32, respectively, and the size of each convolutional kernel was 2. The size of the pooling layer was set to 2, and the stride was set to 1. The ReLU function was selected as the activation function for the convolutional layers. The initial learning rate was set to 0.001, the number of iterations was 200, and Adam was chosen as the optimizer. To accelerate the convergence speed of the network, the batch size was set to 30, and the mean-squared error loss function was used to further reduce the risk of overfitting [39]. In the 1D-CNN quantitative model, except that the size of the convolutional kernels is 3, the stride of the pooling layer is set to 2, and the number of iterations is 300, the remaining parameters are the same as those of the qualitative model.

**Figure 11:**
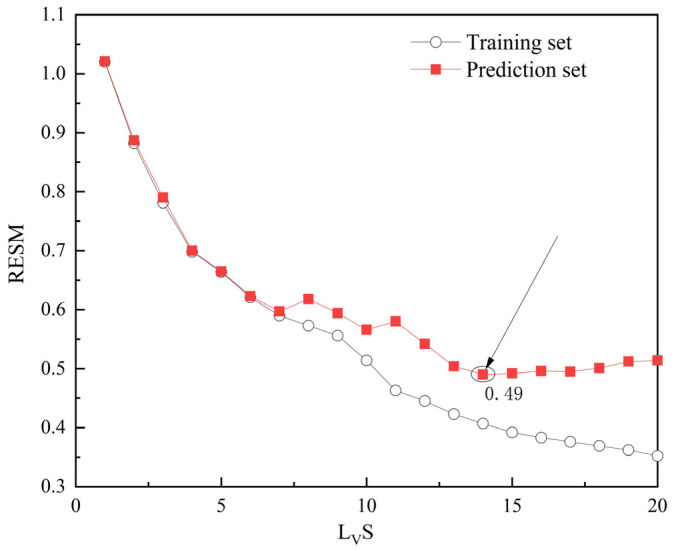
RMSE with different amounts of LVs.

The PLS model used cross-validation to determine the number of LVs. It evaluated the prediction performance under different numbers of LVs. Taking the RMSE of the prediction set as the optimization target, the RMSE corresponding to each number of LVs was calculated, and an RMSE–LVs curve was drawn. The number of LVs was selected when the RMSE reached the minimum value for the first time, and the subsequent fluctuations did not exceed 5% to avoid overfitting. Taking the table grape discrimination model with no GLCM texture features as the input, as an example, its RMSE–LVs curve is shown in Figure 11. When the number of LVs is 14, the RMSE is the lowest with small fluctuations. Therefore, 14 was chosen as the number of LVs for modeling analysis.

The ROI spectrum of table grapes and the corresponding grayscale map texture features corresponding to the wavelength of SPA screening features were input, respectively, and the ELM, CNN, and PLS discriminant models were established by combining the label numbers of table grapes, respectively, and the results are shown in Table 3.

As can be observed from Table 3, the classification models based on fusion features generally performed worse than those using original spectra as inputs. This is primarily because the former retained some variables with low relevance during the extraction of texture features; although the models were optimized, their performance did not improve. The CNN model was the only exception, likely because its automated extraction process targets localized regions, thereby enhancing feature relevance and resulting in superior classification performance compared to the original spectral models. Furthermore, the classification models based on the wavelengths selected by SPA were inferior to the full-band models. This is mainly because the four table grape varieties are extremely similar and exhibit overlapping biochemical characteristics and spectral responses. While eliminating multicollinearity, SPA inevitably discarded some highly subtle spectral information that is crucial for accurate classification.

**Figure 12:**
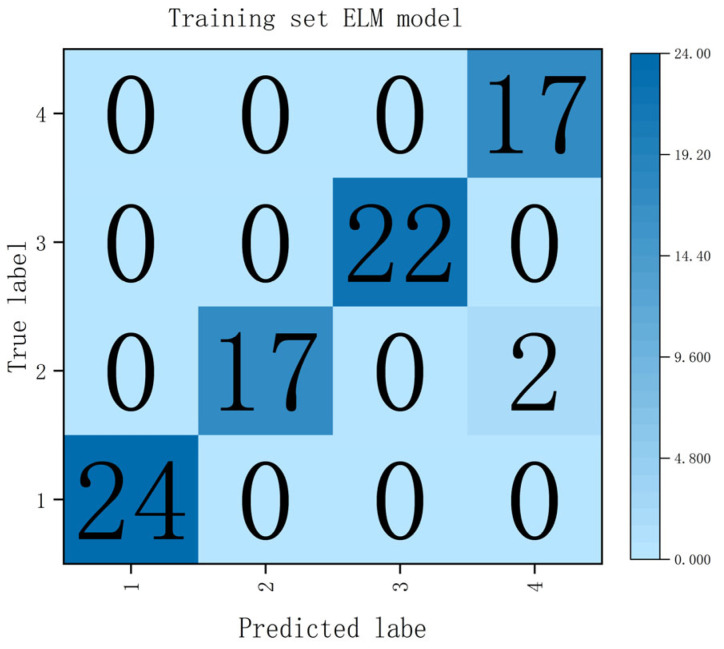
Confusion matrix of the ELM classification model.

Among these, the ELM classification model based on the original spectra demonstrated the optimal performance, achieving the highest classification accuracy of 97.56%, a maximum recall of 97.37%, and a peak F1-score of 97.22%. The confusion matrix for this optimal classification model is illustrated in Figure 12. Conversely, although the PLS classification model based on SPA-selected bands significantly reduced the dimensionality of the input variables and effectively lowered model complexity, it came at the cost of a noticeable decline in classification performance. It yielded the lowest classification accuracy of 54.88%, a minimum recall of 53.73%, and the lowest F1-score of 55.89%. This decline is likely due to the loss of secondary information that is essential for distinguishing between different table grape varieties, thereby weakening the model’s discriminative ability. The CNN classification models, however, consistently maintained good performance across all three types of input features, demonstrating their potential robustness to feature selection.

As observed from the confusion matrix in Figure 12, the ELM model exhibited excellent discriminatory capability across the four table grape varieties. Only two samples of the “Rose” variety were misclassified as “Yongyou”, while all other samples in the prediction set were correctly identified, resulting in a high classification accuracy of 97.56%.

### 3.4. Modeling and Analysis of SSC Prediction for Table Grapes

Based on the qualitative discriminant model inputs, the SSC content of table grapes was linked to the ELM, CNN, and PLS prediction models, respectively, and the results are shown in Table 4.

**Figure 13:**
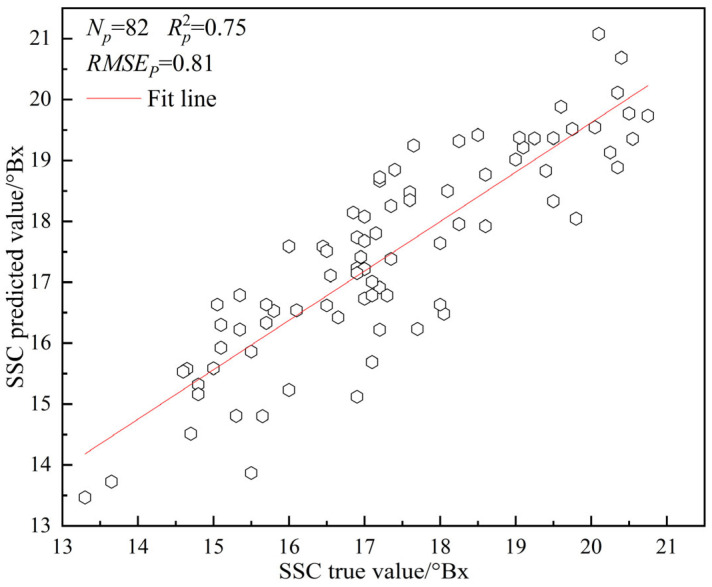
Scatter plot of ELM table grape SSC prediction model based on raw spectrum.

From Table 4, it was observed that SSC prediction models based on original spectra outperformed those utilizing GLCM texture features, which may be attributed to the insufficient representation of SSC information in the GLCM texture features extracted from fresh table grapes. Subsequent attempts to integrate multiple physicochemical indicators for model construction could potentially enhance prediction performance. Among these, the ELM prediction model with 176 input variables demonstrated optimal performance, achieving the highest $R_{p}^{2}$ values of 0.90 and 0.75 for the training and prediction sets, respectively, along with relatively low ${\mathrm{RMSE}}_{p}$ values of 0.71 and 0.81, indicating robust fitting capability and predictive accuracy. In contrast, the ELM model with 48 input variables exhibited the poorest performance, yielding the lowest $R_{p}^{2}$ values of 0.52 and 0.51 for the training and prediction sets, respectively, and the highest ${\mathrm{RMSE}}_{p}$ values of 1.17 and 1.58. CNN-based prediction models displayed intermediate performance under both input variable conditions, potentially due to limited sample size, yet demonstrated notable robustness. The scatter plot illustrating the optimal SSC prediction model for fresh table grapes is presented in Figure 13.

As can be seen from Figure 13, the $R_{p}^{2}$ of the ELM table grape SSC prediction model based on raw spectra was the highest at 0.75, indicating that the real value of table grapes was more closely related to the predicted value, and the RMSEP was the lowest at 0.81, with a small degree of dispersion, and most of the predicted values were distributed near the fitted line, which indicated that the prediction performance was better, and it could satisfy the requirements of detection.

Meanwhile, the best predictive model for SSC in fresh grapes was externally evaluated using 41 samples that were not involved in model construction. Each sample underwent two tests, yielding a total of 82 measurements, with results indicating that the prediction error was within 2° Brix. This further confirms the robustness and reliability of the model in practical applications, providing strong support for the promotion of non-destructive quality assessment of fresh grapes.

Although this study has successfully established models for grape variety classification and SSC prediction, certain challenges remain in practical implementation and promotion. Currently, the research primarily focuses on SSC as the core parameter; however, other quality attributes, such as titratable acidity to sugar ratio and pH, also play important roles in the comprehensive evaluation of grape quality. Additionally, due to the limitation of a single grape harvest season, the model’s adaptability to variable environments is still restricted. In the future, research should further improve the accuracy and generalization ability of the models by introducing multiple quality indicators and expanding the database, thereby enabling the integration of grapes harvested at different times into a unified model framework for in-depth study.

### 3.5. Discussion

The comparative analysis of the qualitative and quantitative models provides a theoretical reference for the application of HSI technology in the non-destructive testing of table grapes.

Firstly, regarding the selection of feature variables, the predictive performance of the full-band models was generally superior to that of the models based on GLCM texture features or SPA-selected wavelengths. This indicates that the physicochemical differences among the four table grape varieties, such as “Shine Muscat”, are relatively subtle. Although GLCM features can reflect the spatial texture information of the peel surface, they struggle to characterize the overtone and combination vibrations of internal hydrogen-containing groups (such as O-H and C-H bonds) that directly determine the SSC. Furthermore, while the SPA algorithm eliminates spectral multicollinearity, it may also filter out certain weak characteristic bands that are crucial for distinguishing closely related varieties.

Secondly, in terms of modeling methods, non-linear models such as ELM and CNN exhibited superior fitting capabilities compared to the traditional linear Partial Least Squares (PLS) method. Due to the complex heterogeneity of the internal tissues of table grapes, the multiple scattering and absorption of photons within the pulp result in a strong non-linear mapping relationship between spectral reflectance and the target physicochemical values. ELM and CNN can more effectively extract and fit these non-linear features, thereby reducing prediction errors. Conversely, constrained by linear assumptions, PLS exhibits certain limitations when handling such complex multivariate relationships.

Finally, although this study has successfully demonstrated the potential of combining HSI with non-linear algorithms for non-destructive testing as an alternative to traditional destructive physicochemical analysis, certain challenges remain in its practical implementation and promotion. Current research primarily focuses on SSC as the core parameter; however, other quality indicators, such as the sugar–acid ratio and pH, also play crucial roles in the comprehensive evaluation of table grape quality. Additionally, limited by the single harvest season of the table grapes, the current models exhibit restricted adaptability to variable environments and growth conditions. Future research should further enhance the accuracy and generalization capabilities of the models. By introducing multiple quality indicators and expanding the database, future studies should aim to integrate table grapes from different harvest periods into a unified modeling framework.

## 4. Conclusions

Addressing the limitations of singular methodologies in assessing table grape quality, this study utilized HSI for a combined spectral and image analysis to determine table grape varieties and their SSC.

(1) The investigation into the SSC levels of four distinct table grape varieties revealed that the “Yongyou” cultivar had the highest average SSC, recorded at 19.65° Brix, whereas the “Xiahei” variety exhibited the lowest SSC at 16.23° Brix. Furthermore, no significant difference in SSC was identified between the “Jumbo” and “Xiahei” varieties; however, significant differences were observed between the “Rose” and “Yongyou” varieties compared to the others.

(2) Two distinct models were established for the classification of table grape varieties, employing the original spectral data and GLCM texture features as input parameters. The results indicated that the ELM model, utilizing the original spectral data, achieved the highest classification accuracy of 97.56%. Conversely, the PLS model, relying on GLCM texture features, demonstrated the least effectiveness, with a minimum classification accuracy of 56.10%.

(3) In the quantitative assessment of SSC across the four table grape varieties, the ELM prediction model, when supplied with 176 input variables, exhibited robust predictive performance, achieving an $R_{p}^{2}$ value of 0.75 and a minimum ${\mathrm{RMSE}}_{p}$ of 0.81. Conversely, the ELM model employing 48 input variables exhibited diminished performance, with a minimum $R_{p}^{2}$ of 0.51 and a maximum ${\mathrm{RMSE}}_{p}$ of 1.58.

(4) Regardless of qualitative or quantitative analysis, CNN exhibited robust performance and potential. These findings provide a theoretical foundation for establishing a combined HSI spectral-image detection method for table grape quality.

## Figures and Tables

**Table 1:** Results of data set division of 4 kinds of grapes.

Variety	Variety (Unit: Pieces)				Totality (Unit: Pieces)
	Jumbo	Rose	Xiahei	Yongyou	
Training set	63	63	71	67	263
Prediction set	24	19	22	17	82
Label number	1	2	3	4	—

**Table 2:** Results of difference analysis of SSC content in 4 table grapes.

Variety	Quantity	The Mean ± Standard Deviation	Maximum	Minimum	F	*p*
Jumbo	87	16.43 ± 1.05 ^c^	18.30	14.25	230.48	<0.001
Rose	81	18.04 ± 0.77 ^b^	20.10	16.60		
Xiahei	93	16.23 ± 1.15 ^c^	18.40	13.30		
Yongyou	84	19.65 ± 0.86 ^a^	21.30	17.20		

Note: Data are expressed as mean ± standard deviation. Statistical analysis was performed using One-Way ANOVA via SPSS software. Different superscript letters (a–c) within the same column indicate statistically significant differences between the grape varieties at the *p* < 0.05 level based on post-hoc multiple comparisons.

**Table 3:** Results of table grape discrimination model.

Method	Input Features	Number of Variables	Accuracy/%	Recall/%	F1-Score/%
ELM	Full band	176	97.56	97.37	97.22
	SPA band	10	73.17	71.60	72.63
	Fusion	48	64.63	63.35	62.91
CNN	Full band	176	91.46	90.50	90.76
	SPA band	10	91.46	90.48	90.27
	Fusion	48	95.12	94.92	94.61
PLS	Full band	176	64.63	64.49	64.83
	SPA band	10	54.88	53.73	55.89
	Fusion	48	56.10	55.50	56.38

Fusion: GLCM texture features were extracted in four directions from the images of the 12 combined bands, which were formed by merging the SPA-selected bands with the sensitive bands identified from the average ROI spectra.

**Table 4:** Results of SSC prediction model for table grapes.

Method	Number of Variables	$R_{c}^{2}$	RMSEC	$R_{p}^{2}$	RMSEP
ELM	176	0.90	0.71	0.75	0.81
	48	0.52	1.17	0.51	1.58
CNN	176	0.88	0.32	0.74	0.91
	48	0.73	0.85	0.64	1.07
PLS	176	0.81	0.72	0.74	0.92
	48	0.59	1.06	0.60	1.13

## Data Availability

The datasets analyzed during the current study are available from the corresponding author on reasonable request.

## Abbreviations

The following abbreviations are used in this manuscript:

SSC	Soluble Solids Content
GLCM	Gray-Level Co-occurrence Matrix
SPA	Successive Projections Algorithm
ELM	Extreme Learning Machine
CNN	Convolutional Neural Network
PLS	Partial Least Squares
NDT	Non-destructive testing
HSI	Hyperspectral imaging
$R^{2}$	Coefficient of determination
RMSE	Root mean square error
LVs	Latent variables
ROI	Region of interest
